# Molecular characterization of sessile serrated adenoma/polyps with dysplasia/carcinoma based on immunohistochemistry, next-generation sequencing, and microsatellite instability testing: a case series study

**DOI:** 10.1186/s13000-018-0771-3

**Published:** 2018-11-20

**Authors:** Takashi Murakami, Yoichi Akazawa, Noboru Yatagai, Takafumi Hiromoto, Noriko Sasahara, Tsuyoshi Saito, Naoto Sakamoto, Akihito Nagahara, Takashi Yao

**Affiliations:** 1 Department of Gastroenterology, Juntendo University School of Medicine, 2-1-1 Hongo, Bunkyo-ku, Tokyo, 113-8421 Japan; 2 Department of Human Pathology, Juntendo University School of Medicine, Tokyo, Japan

**Keywords:** Sessile serrated adenoma/polyp, Colorectal carcinoma, MLH1, *FBXW7*, *TP53*, Microsatellite instability

## Abstract

**Background:**

Colorectal sessile serrated adenoma/polyps (SSA/Ps) are considered early precursor lesions in the serrated neoplasia pathway. Recent studies have shown associations of SSA/Ps with lost MLH1 expression, a CpG island methylator phenotype, and *BRAF* mutations. However, the molecular biological features of SSA/Ps with early neoplastic progression have not yet been fully elucidated, owing to the rarity of cases of SSA/P with advanced histology such as cytologic dysplasia or invasive carcinoma. In this study, we aimed to elucidate the molecular biological features of SSA/Ps with dysplasia/carcinoma, representing relatively early stages of the serrated neoplasia pathway.

**Methods:**

We performed immunostaining for β-catenin, MLH1, and mucins (e.g., MUC2, MUC5AC, MUC6, and CD10); targeted next-generation sequencing; and microsatellite instability (MSI) testing in 8 SSA/P lesions comprised of 4 SSA/Ps with high-grade dysplasia and 4 SSA/Ps with submucosal carcinoma.

**Results:**

Lost MLH1 expression was found in 5 cases. All lesions studied were positive for nuclear β-catenin expression. Regarding phenotypic mucin expression, all lesions were positive for MUC2, but negative for CD10. MUC5AC and MUC6 positivity was observed in 7 cases. Genetically, the most frequently mutated gene was *BRAF* (7 cases), and other mutations were detected in *FBXW7* (3 cases); *TP53* (2 cases), and *KIT*, *PTEN*, *SMAD4*, and *SMARCB1* (1 case each). Furthermore, 4 of 8 lesions were MSI-high and the remaining 4 lesions were microsatellite-stable (MSS). Interestingly, all 4 MSI-high lesions displayed MLH1 loss, 3 of which harbored a *FBXW7* mutation, but not a *TP53* mutation. However, 2 MSS lesions harbored a *TP53* mutation, although none harbored a *FBXW7* mutation.

**Conclusions:**

SSA/Ps with dysplasia/carcinoma frequently harbored *BRAF* mutations. Activation of the WNT/β-catenin signaling pathway may facilitate the development of dysplasia in SSA/Ps and progression to carcinoma. Furthermore, our results suggested that these lesions might be associated with both MSI-high and MSS colorectal cancer, which might be distinguished by distinct molecular biological features such as lost MLH1 expression, *FBXW7* mutations, and *TP53* mutations.

## Background

In 2003, Torlakovic et al. [1] reported evidence of abnormal proliferation in colorectal serrated polyps that superficially resembled hyperplastic polyps (but that could be distinguished histologically based on their abnormal architectural features) and introduced the terms “sessile serrated polyp” and “sessile serrated adenoma” to describe their observations. Currently, this category is designated as “sessile serrated adenoma/polyp (SSA/P),” as recommended by the World Health Organization [2]. SSA/P is considered as an early precursor lesion in the serrated neoplasia pathway, which largely results in colorectal carcinomas with high levels of microsatellite instability (MSI-high) [3–5]. Recent studies have shown associations of SSA/Ps and those with dysplasia/carcinoma with DNA methylation or lost protein expression of DNA-repair genes (i.e., *MLH1*) [1, 4, 6–9], a CpG island methylator phenotype [3, 4, 6, 7], and *BRAF* mutations [3, 4, 6–13]. This pathway is thought to be distinct from the conventional adenoma-carcinoma pathway, where adenomas progress to invasive colorectal carcinomas through the influence of several genetic alterations including adenomatous polyposis coli (*APC*) and *KRAS* mutations [4, 6, 10, 11, 14, 15].

Recently, targeted next-generation sequencing (NGS) has shown unprecedented potential for detecting underlying changes in the genetic architecture of cancer in a comprehensive and economically feasible manner, and the development of its platforms has enabled comprehensive analysis of genetic alterations in tumors [16, 17]. A comprehensive understanding of the genetic alterations associated with cancer could improve the molecular biological classifications of tumors and identify effective molecularly targeted therapies.

At present, the molecular biological features underlying the development of colorectal serrated neoplasia remain unclear. Hence, the aim of this study was to employ NGS to elucidate the molecular biological features of SSA/Ps with dysplasia/carcinoma, representing relatively early stages of the serrated neoplasia pathway.

## Methods

### Patients and materials

Eight colorectal lesions (from 8 different patients) were resected endoscopically or surgically at Juntendo University Hospital between 2014 and 2016, and used in this study. These lesions comprised 4 SSA/Ps with high-grade dysplasia and 4 SSA/Ps with submucosal carcinoma. The diagnosis of SSA/P was based on the following criteria described by Torlakovic et al. [1]: the presence of serration at the base of crypts, irregularly dilated crypts, irregularly branching crypts, and horizontally and/or laterally arranged basal crypts. The histologic features of the high-grade dysplasia were assessed as described previously [2, 12], as follows: a tubular, tubulovillous, or fused glandular pattern (mimicking conventional adenomatous high-grade dysplasia) or a serrated glandular pattern (preserving the serrated or saw-toothed structure with infolding of the crypt epithelium), which consisted of cuboidal and eosinophilic dysplastic cells with substantially larger nuclei and irregular thickening of the nuclear membrane (i.e. ‘serrated-type’ high-grade dysplasia). Submucosal carcinoma was defined as an obvious epithelial neoplasm histologically atypical enough to be diagnosed as at least high-grade dysplasia that was invading the muscularis mucosa into the submucosa. The inclusion criteria for SSA/P with high-grade dysplasia or with submucosal carcinoma included a component of ordinary SSA/P visible at the lesion edge that comprised at least 3 crypts, with an SSA/P-type histology required in 1 crypt. All samples were reviewed independently by 2 authors (TY and TM).

**Fig. 1:**
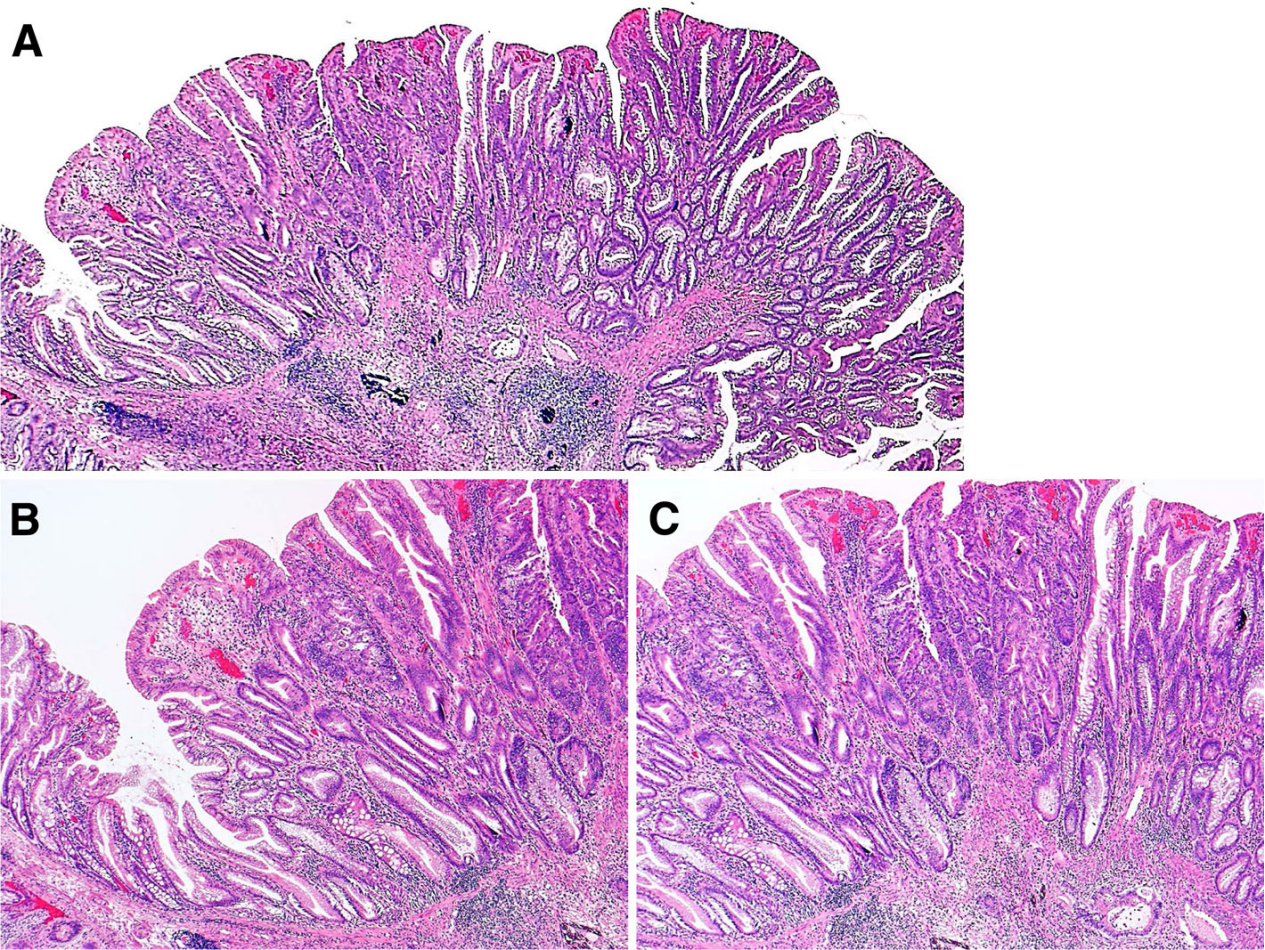
Typical morphologies in a representative case of sessile serrated adenoma/polyp (SSA/P) with submucosal carcinoma (Case #6). **a** Dilated crypts with deep serration was seen on both sides of the lesion, and high-grade dysplasia with submucosal invasion was seen in the middle. **b** High-power field of (**a**) (the left side). Crypts with a serrated architecture included those that were irregularly dilated, irregularly branched, and horizontally arranged (basal), corresponding to SSA/P. **c** High-power field of (**a**) (the middle). Well-differentiated tubular adenocarcinoma was observed invading the submucosa. Adjacent SSA/P areas were observed towards both sides of the panel. An abrupt transition was evident between the 2 adjacent regions

Typical morphologies in representative cases of SSA/P with submucosal carcinoma are shown in Fig. 1.

### Clinicopathological analysis

For each patient, we recorded the age, sex, tumor location (proximal colon defined as proximal to the splenic flexure, remaining colon defined as distal), macroscopic type, whole tumor size (including both high-grade dysplasia or invasive carcinoma and in situ SSA/P), histological type (based on the World Health Organization’s histological classification of tumors), depth of submucosal invasion, lymphovascular invasion, and lymph node metastasis.

### Immunohistochemical analysis

Four-micrometer-thick serial tissue sections prepared from formalin-fixed and paraffin-embedded tissues were subjected to immunohistochemistry. Staining was performed using a Dako EnVision Kit with antibodies against MLH1 (ab14206; 1:50 dilution; Abcam, CA, USA) and β-catenin (clone 14, 1:200 dilution; BD Bioscience, San Diego, CA, USA). Tumor phenotypes were evaluated by immunostaining with antibodies from Novocastra (Newcastle upon Tyne, UK; 1:100 dilution) against MUC2 (NCL-MUC-2), MUC5AC (NCL-MUC-5 AC), MUC6 (NCL-MUC-6), and CD10 (NCL-CD10–270). Appropriate positive and negative controls were used for each antibody.

Loss of MLH1 expression was noted when one or more clusters of tumor cells (minimal, focal, or multifocal) or all malignant cells showed no nuclear staining, compared with positive nuclear staining in normal epithelial cells and lymphocytes [1]. Expression of β-catenin, which generally showed an inverse relationship between membranous and nuclear (with cytoplasmic) reactivity, was only evaluated in terms of nuclear localization in this study. Nuclear β-catenin expression was considered as positive when distinct and strong nuclear staining was observed in over 5% of the tumor cells [9]. Membrane staining for CD10, and cytoplasmic staining for MUC2, MUC5AC, and MUC6 were judged as positive, when over 5% of tumor cells showed a positive reaction for each marker. The immunohistochemical staining results were evaluated by 2 authors (TY and TM).

### DNA extraction

Genomic DNA was extracted from 5 formalin-fixed paraffin-embedded sections (10-mm-thick) using a QIAamp DNA FFPE Tissue Kit (Qiagen GmbH, Hilden, Germany), according to the manufacturer’s instructions. Sections were stained lightly with hematoxylin, and only the dysplastic or carcinomatous areas in the lesions were microdissected with direct observation of the tissue under a light microscope. The DNA quality and integrity were checked spectrophotometrically.

### Targeted NGS

The 50-gene Ion AmpliSeq Cancer Hotspot Panel v2 (Life Technologies) was used with the Ion-Torrent ™ Personal Genome Machine platform (Life Technologies, Foster city, CA, USA) in all experiments. This panel is designed to amplify 207 amplicons covering approximately 2800 mutations deposited in the COSMIC database from 50 oncogenes and tumor-suppressor genes commonly mutated in human cancers (*ABL1*, *AKT1*, *ALK*, *APC*, *ATM*, *BRAF*, *CDH1*, *CDKN2A*, *CSF1R*, *CTNNB1*, *EGFR*, *ERBB2*, *ERBB4*, *EZH2*, *FBXW7*, *FGFR1*, *FGFR2*, *FGFR3*, *FLT3*, *GNA11*, *GNAS*, *GNAQ*, *HNF1A*, *HRAS*, *IDH1*, *IDH2*, *JAK2*, *JAK3*, *KDR*, *KIT*, *KRAS*, *MET*, *MLH1*, *MPL*, *NOTCH1*, *NPM1*, *NRAS*, *PDGFRA*, *PIK3CA*, *PTEN*, *PTPN11*, *RB1*, *RET*, *SMAD4*, *SMARCB1*, *SMO*, *SRC*, *STK11*, *TP53*, and *VHL*). The Ion AmpliSeq Library Kit, version 2.0 (Life Technologies) was used to amplify 10 ng of DNA according to the manufacturer’s instructions. Sequencing beads were templated and enriched using the Hi-Q Template OT2 200 Kit, and sequencing was performed on 318v2 chips using the Hi-Q Sequencing Kit (Life Technologies) according to the manufacturer’s protocols. Signal processing, mapping, and quality control were performed with Torrent Suite, v.5.0 (Life Technologies). Sequence variants were called using Ion Reporter, v5.2 using the AmpliSeq CHPv2 single-sample workflow and default settings. Variants were categorized according to whether they comprised a nonsynonymous or frameshift mutation, or stop codon in the exonic region. The limit of detection was a 5% mutational allelic frequency at 500 × coverage or a 3% mutational allelic frequency at 1000 × coverage for each tested region. The minimum coverage depth was 500 × .

### MSI testing

DNA extracted from microdissected paraffin-embedded tumor sections and non-neoplastic tissues was analyzed by a polymerase chain reaction–based method, followed by capillary electrophoretic detection. MSI detection was performed using a panel of 5 mononucleotide microsatellite markers (BAT-25, BAT-26, NR-21, NR-24, and MONO-27). In accordance with National Cancer Institute guidelines, MSI at 2 loci or more was defined as MSI-high, instability at a single locus was defined as low levels of MSI (MSI-low), and no instability at any of the loci tested was defined as microsatellite-stable (MSS).

## Results

### Clinicopathological analysis

The detailed clinicopathological findings of the 8 SSA/P lesions are shown in Table 1. This study included 2 male and 6 female SSA/P patients, with ages ranging from 61 to 79 years (mean 69 years). Six lesions were within the proximal colon, and the remaining 2 lesions were in the distal colon. Macroscopically, sessile morphology was frequently observed among the studied SSA/P lesions. The mean tumor size was 18 mm (range: 10 to 31 mm).

**Table 1:** Summary of clinicopathological features in each case of sessile serrated adenoma/polyp (SSA/P) with dysplasia and invasive adenocarcinoma

Case No.	Age	Sex	Location	Macroscopic type	Size (mm)	Histological type	Depth of invasion (μm)	Mucinous component	Lymphatic invasion	Vascular invasion	Lymph node metastasis	Removal method
1	67	F	A	Sessile	10	HGD	Mucosa	–	–	–	–	EMR
2	69	M	S	Semipedunculated	10	HGD	Mucosa	–	–	–	–	EMR
3	79	F	C	Sessile	14	HGD	Mucosa	–	–	–	–	EMR
4	61	F	A	Sessile	18	HGD	Mucosa	–	–	–	–	ESD
5	61	M	D	Sessile	31	Well-ACA	Submucosa (400)	–	–	–	–	ESD + OPE
6	75	F	A	Sessile	15	Well-ACA	Submucosa (1100)	–	–	–	–	EMR + OPE
7	73	F	A	Sessile	25	Mod-ACA	Submucosa (2000)	–	+	–	+	OPE
8	65	F	T	Sessile	19	Mod-ACA	Submucosa (4000)	+	–	–	–	OPE

*M* Male, *F* Female, *C* Cecum, *A* Ascending colon, *T* Transverse colon, *D* Descending colon, *S* Sigmoid colon, *HGD* High-grade dysplasia, *Well-ACA* Well-differentiated adenocarcinoma, *Mod-ACA* Moderately-differentiated adenocarcinoma, *EMR* Endoscopic mucosal resection, *ESD* Endoscopic submucosal dissection, *OPE* Operation, + present; − absent

Histologically, 4 lesions were high-grade dysplasias, and the other 4 lesions were well-to-moderately differentiated tubular adenocarcinomas invading into the submucosa, of which 1 lesion was accompanied with a mucinous component in the submucosa. Lymphatic invasion and lymph node metastasis were found in 1 case of SSA/P with carcinoma.

### Immunohistochemical analysis

**Fig. 2:**
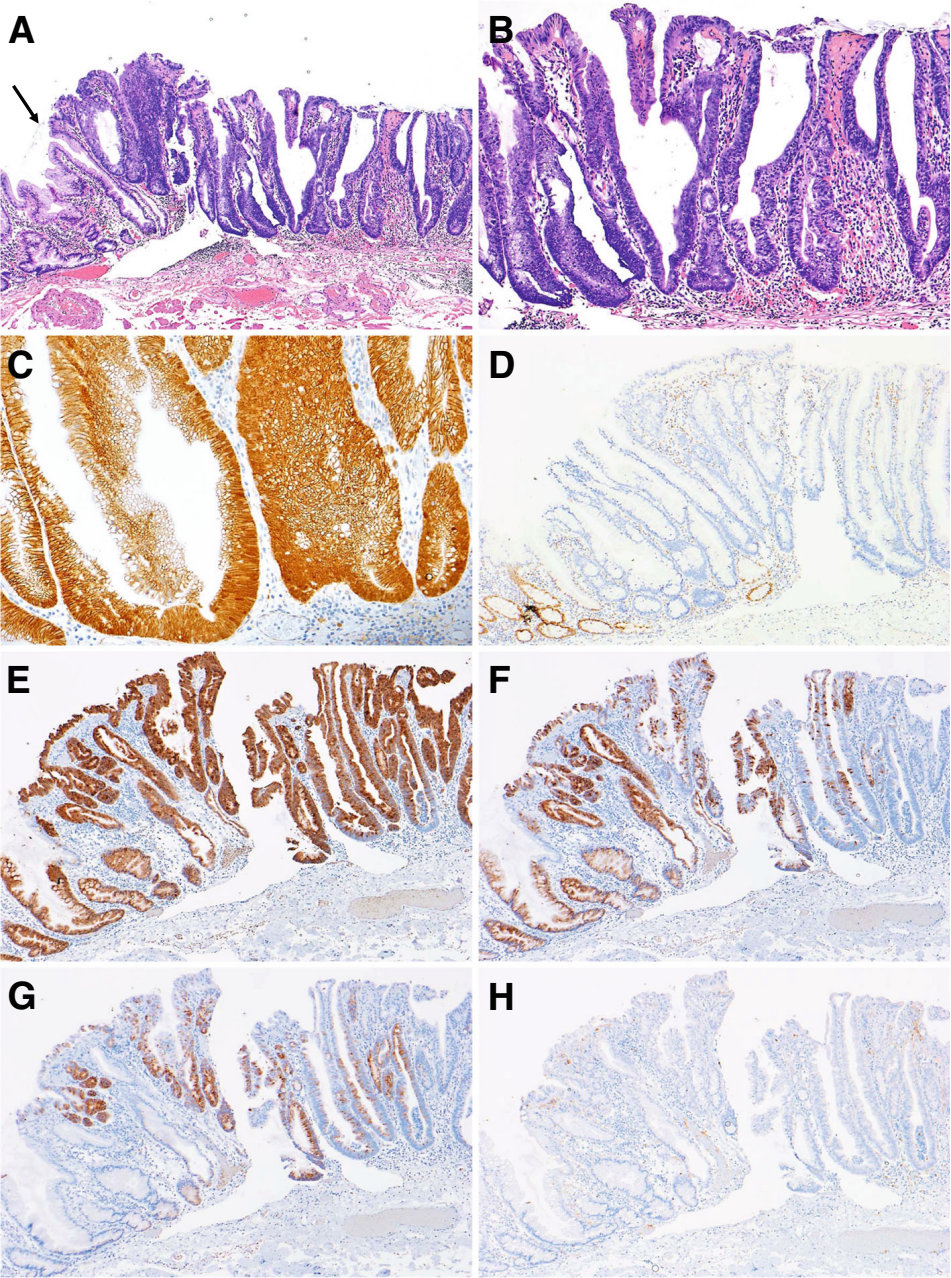
Immunohistochemical staining in a representative case of sessile serrated adenoma/polyp (SSA/P) with dysplasia (Case #2). **a** Dilated crypts and deep serration, corresponding to SSA/P, were seen on the left side, and high-grade dysplasia without submucosal invasion was seen on the right side. An abrupt transition was seen between the 2 adjacent regions (arrow). **b** High-power field of (**a**) (the right side). A view shows high-grade dysplasia without submucosal invasion, which was pathologically consistent with SSA/P with cytologic dysplasia. The lesion showed nuclear β-catenin expression (**c**) and a loss of MLH1 expression (**d**). Regarding phenotypic mucin expression, the lesion was positive for MUC2 (**e**), MUC5AC (**f**), and MUC6 (**g**) expression, and negative for CD10 (H) expression

Loss of MLH1 expression was found in 5 cases. Without exception, all lesions were positive for nuclear β-catenin expression. Regarding phenotypic mucin expression, all lesions were positive for MUC2, but negative for CD10. MUC5AC and MUC6 were positive in 7 of 8 cases (88%). Immunohistochemical staining of these proteins in a representative case is illustrated in Fig. 2.

### Mutation analysis

**Fig. 3:**
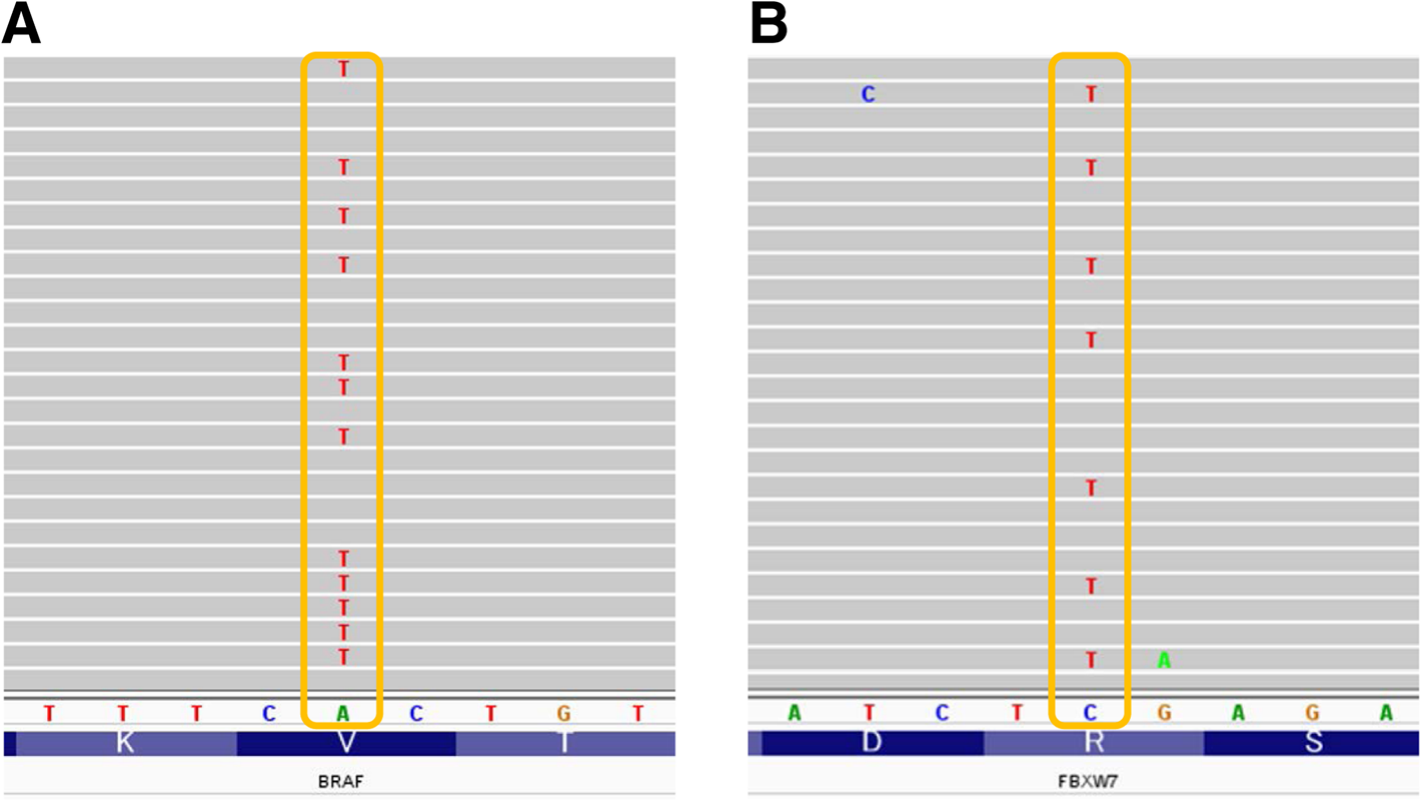
Representative examples of mutations detected by next-generation sequencing. **a** Case #1, *BRAF* mutation, V600E (c.1799 T > A). **b** Case #3, *FBXW7* mutation, R479Q (c.1436 G > A)

All SSA/P lesions had acceptable DNA integrity (the average RQ value was 0.18) and were subjected to NGS. The genetic alterations identified by NGS in 8 SSA/P lesions are summarized in Table 2. The most frequently mutated gene was *BRAF* (7 of 8; 88%), and the other detected mutations were in *FBXW7* (3 of 8; 38%), *TP53* (2 of 8; 25%), *KIT* (1 of 8; 13%), *PTEN* (1 of 8; 13%), *SMAD4* (1 of 8; 13%), and *SMARCB1* (1 of 8; 13%). The only *BRAF* mutation found was V600E (c.1799 T > A). The *FBXW7* mutations found were R278X (c.832 C > T), R479Q (c.1436 G > A), and Q508Q (c.1524 A > G). Examples of data analysis using the Integrative Genomics Viewer ™ software are shown in Fig. 3.

**Table 2:** Summary of molecular biological features in each case of sessile serrated adenoma/polyp (SSA/P) with dysplasia and invasive adenocarcinoma

Case No.	Group	Immunohistochemistry						Next generation sequencing analysis (Gene mutations)							MSI analysis
		MLH1 loss	β-catenin	MUC2	MUC5AC	MUC6	CD10	*BRAF*	*FBXW7*	*KIT*	*PTEN*	*SMAD4*	*SMARCB1*	*TP53*	
1	Dysplasia	–	+	+	+	+	–	+	–	–	–	–	–	+	MSS
2	Dysplasia	+	+	+	+	+	–	+	–	–	–	–	–	–	MSI-high
3	Dysplasia	+	+	+	+	+	–	+	+	+	–	+	–	–	MSI-high
4	Dysplasia	–	+	+	+	+	–	+	–	–	+	–	+	–	MSS
5	Carcinoma	+	+	+	+	+	–	+	–	–	–	–	–	–	MSS
6	Carcinoma	–	+	+	–	–	–	+	–	–	–	–	–	+	MSS
7	Carcinoma	+	+	+	+	+	–	–	+	–	–	–	–	–	MSI-high
8	Carcinoma	+	+	+	+	+	–	+	+	–	–	–	–	–	MSI-high

Dysplasia, SSA/P with high-grade dysplasia; Carcinoma, SSA/P with submucosal carcinoma; MSI-high, Microsatellite instability-high; MSS, Microsatellite-stable; +, present; −, absent

### MSI testing

Of the 8 SSA/P lesions, 4 lesions (including 2 SSA/Ps with dysplasia and 2 SSA/Ps with carcinoma) were MSI-high and the remaining 4 lesions were MSS (Table 2).

### Associations between immunohistochemistry, mutation analysis, and MSI

All 4 MSI-high SSA/P lesions showed a loss of MLH1 expression, and 3 of those lesions harbored an *FBXW7* mutation, but not a *TP53* mutation. In contrast, among the SSA/P lesion with MSS, only 1 showed loss of MLH1 expression, 2 cases harbored a *TP53* mutation, and no cases harbored an *FBXW7* mutation.

## Discussion

Rare occurrences of *BRAF* mutations have been documented for conventional colorectal carcinomas, although they are frequent in dysplasia/carcinoma arising from SSA/Ps (50–90%) [3, 4, 9, 10, 12, 13]. In this study, *BRAF* mutations were detected in 7 of 8 SSA/P lesions, in accordance with previous reports [3, 4, 9, 10, 12, 13]. *BRAF* mutations result in activation of the RAS-RAF-MAPK pathway, and therefore our findings and previous reports [3, 4, 9, 10, 12, 13] suggest that those signaling pathways might be activated in serrated neoplasia.

We investigated the core protein-expression levels of MUC2, MUC5AC, MUC6, and CD10 because altered expressions of these proteins may be significantly correlated with the biological behavior of colorectal carcinoma and, possibly its prognosis. In this study, MUC2, MUC5AC, and MUC6 expression was frequently positive in the serrated lesions. MUC2 is a goblet cell-type mucin predominantly expressed in the colon. In contrast, MUC5AC and MUC6 are two gastric type mucins that are expressed in the surface foveolar epithelium and deep antral/pyloric glands, respectively, but not normally in colonic mucosa. MUC5AC and MUC6 expression have been strongly associated with tumorigenesis via the serrated neoplasia pathway [18–20], in agreement with our current findings.

The WNT signaling pathway involves β-catenin and plays a crucial role in the development of colorectal carcinomas through a conventional adenoma-carcinoma progression [21]. Several previous reports including our own [8, 9, 12, 13, 22] have also demonstrated that nuclear β-catenin accumulation is common in SSA/Ps with dysplasia and invasive carcinoma, but not in those without dysplasia. This observation implies that activation of the WNT/β-catenin signaling pathway is involved in the development of dysplasia in SSA/Ps and progression to carcinoma. Although *APC* and *CTNNB1* mutations are major causes of WNT/β-catenin signaling activation in conventional-type adenomas, these genetic alterations are absent in SSA/Ps [13]. In this study, all serrated lesions examined also displayed nuclear β-catenin immunoreactivity, whereas no mutations in WNT/β-catenin signaling-associated genes (such as *APC* and *CTNNB1*) were found in the studied lesions. Previously, we reported that activation of the WNT/β-catenin signaling pathway might be associated with methylation of the associated genes, including *SFRP4*, *MCC*, and *AXIN2* [9]. This association could explain at least some of the discrepancies between β-catenin immunoreactivity and the lack of associated gene mutations.

The MSI phenotype has been regarded as a main subtype of colorectal cancers. Sporadic colorectal cancers with the MSI-high phenotype account for approximately 3–15% of all colorectal cancers [23]. MSI is a unique molecular alteration induced by deficiencies in the DNA-mismatch repair system and is characterized by unstable (variable length) microsatellites, a type of simple DNA sequence repeat. Some previous studies have shown that serrated neoplasia is associated with MSI-high colorectal carcinomas, which is accompanied with DNA methylation or loss of protein expression of DNA-repair genes such as *MLH1* [3–5]. In our study, all 4 MSI-high SSA/P lesions showed a loss of MLH1 expression, in accordance with previous reports [3–5].

A recent report by the Cancer Genome Atlas Project elucidated the molecular landscape of colorectal cancers and revealed that the hypermutated phenotype mainly overlaps with MSI status in colorectal cancers [24]. Many types of genetic mutations can occur in MSI-high colorectal cancers. Indeed, a previous report showed that mutations in various genes, including the tumor-suppressor gene *PTEN* and the oncogene *PIK3CA*, were caused by the instability of microsatellites in MSI-high colorectal cancers [25]. In this study, no MSI-high lesions harbored *PTEN* or *PIK3CA* mutations. However, it is interesting that 3 of 4 MSI-high lesions harbored an *FBXW7* mutation, whereas no MSS lesions harbored an *FBXW7* mutation. *FBXW7* is a tumor-suppressor gene located on human chromosome 4q that encodes a substrate-recognition component of SKP1–Cullin1–F-box protein-ubiquitin E3 ligase complexes [26]. These specific E3 ligase complexes negatively regulate the intracellular abundance of an expanding list of key oncogenic proteins such as cyclin E [27], c-JUN [28, 29], c-MYC [30, 31], MCL1 (myeloid cell leukemia 1) [32, 33], NOTCH [34–36], AURKA (aurora kinase A) [37, 38], KLF5 (Krüppel-like factor 5) [39], mTOR [40], and TGIF1 [41]. Therefore, the loss of FBXW7 function results in accumulation of its substrates, which leads to oncogenesis and progression of multiple cancers including colorectal cancers [42, 43]. A study of over 500 primary tumors of diverse tissue origins suggested that *FBXW7* mutations occurred in approximately 6% of all evaluated tumors. Of these, the most commonly affected tumors were cholangiocarcinoma (35%), T-cell acute lymphocytic leukemia (31%), endometrial cancer (9%), and gastric cancer (6%) [43]. *FBXW7* has also consistently been identified as one of the most commonly mutated genes in colorectal cancer, being observed in 6 to 10% of all cases [43, 44]. Furthermore Chang et al. reported that *FBXW7-*mutant colorectal cancers had significant associations with MSI-high tumors in a large-scale study of 1519 cases [45]. Our findings and those previous reports indicated that *FBXW7* mutations might potentially be involved in the progression of MSI-high serrated lesions.

**Fig. 4:**
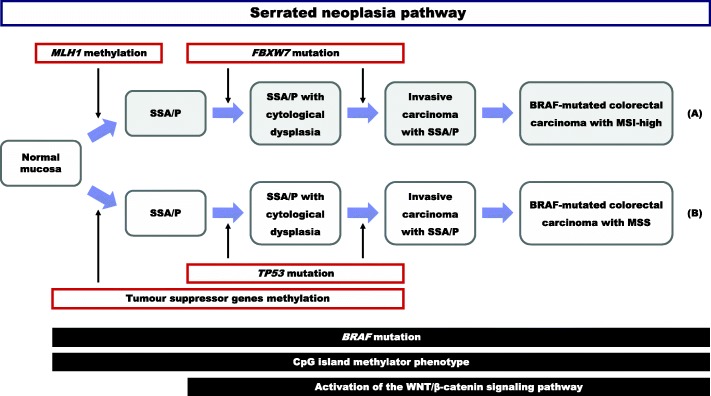
Schematic representation of differences in the molecular biological expressions and genetic alterations in the serrated neoplasia pathway. Sessile serrated adenoma/polyp (SSA/P) is an early precursor lesion in the serrated neoplasia pathway that progresses to cytological dysplasia and results in *BRAF*-mutated colorectal carcinomas that are commonly high levels of microsatellite instability (MSI-high) (diagram A) or microsatellite-stable (MSS) (diagram B). Both pathways are associated with a CpG island methylator phenotype and WNT/β-catenin signaling activation. **a** The upper arm, driven by *BRAF* mutation and *MLH1* methylation, indicates progression to *BRAF*-mutated MSI-high carcinoma. *FBXW7* mutations are potentially involved in progression of this pathway. **b** The lower arm, driven by *BRAF* mutation and methylation of unspecified tumor-suppressor genes, involves progression to *BRAF*-mutated MSS carcinoma. *TP53* mutations are potentially involved in progression of this pathway

Activation of the second arm of the serrated neoplasia pathway, also driven by CpG island methylation of unspecified tumor-suppressor genes but not DNA-repair genes, may indicate progression to *BRAF*-mutant MSS carcinoma [46, 47]. Furthermore, as reported in earlier works [48–50], a strong inverse correlation was found between *TP53* alterations and the MSI phenotype. In this study, 2 of 4 lesions with MSS harbored a *TP53* mutation, whereas no MSI-high lesions harbored a *TP53* mutation, similar to previous observations [48–50]. The *TP53* gene encodes a tumor-suppressor protein containing transcriptional-activation, DNA-binding, and oligomerization domains. The encoded protein responds to diverse cellular stresses to regulate the expression of target genes, thereby inducing cell cycle arrest, apoptosis, senescence, DNA repair, or changes in metabolism [51]. Mutations in the *TP53* gene are reportedly associated with a variety of human cancers, including colon, breast, lung, and brain cancers [51]. Our findings and those previous reports indicated that *TP53* mutations are potentially involved in the progression of MSS serrated lesions. A schematic depiction of differences in the expression of key proteins and genetic alterations in the serrated neoplasia pathway is shown in Fig. 4.

Our study had a major limitation; the sample size was very small. A major impediment to investigating dysplasia/carcinoma arising in SSA/P is the rarity of this lesion. Additionally, a sufficient amount of specimen is necessary for NGS analysis, which limited the lesions that could be studied to those of 10 mm in diameter or more. These limitations lowered the statistical power of this study. Particularly, although the high rate of *FBXW7* or *TP53* mutations in the studied lesions is important, the possibility that this result was a chance occurrence cannot be denied, considering that only 2 or 3 cases of the studied lesions harbored *TP53* or *FBXW7* mutations, respectively.

To the best of our knowledge, few comprehensive genetic studies have been conducted in SSA/Ps with dysplasia and invasive carcinoma. Our results revealed mutations in *BRAF*, *FBXW7*, *TP53*, *KIT*, *PTEN*, *SMAD4*, and *SMARCB1*. Furthermore, it is very interesting that all MSI-high lesions might be associated with MLH1 expression loss and mutation of *FBXW7*, but not *TP53*. Our results may help clarify the detailed mechanism of serrated neoplasia development. A comprehensive understanding of genetic alterations associated with the serrated neoplasia pathway could help identify effective molecularly targeted therapies.

## Conclusions

In conclusion, colorectal SSA/Ps with dysplasia and invasive carcinoma frequently harbored *BRAF* mutations and showed nuclear β-catenin expression. Furthermore, these lesions might not only be associated with MSI-high colorectal cancer, but also MSS, and MSI-high and MSS serrated lesions might have distinct genetic features (such as *FBXW7* and *TP53* mutations). *BRAF*-mutant MSS colon carcinomas are particularly important because they have a dismal prognosis and an aggressive clinical course with adverse histologic features, such as lymphatic and perineural invasion and high tumor budding [52, 53]. Further investigations are required to elucidate molecular biological characteristics in the serrated neoplasia pathway in greater detail.

## Abbreviations

*APC*: Adenomatous polyposis coli
MSI: Microsatellite instability
MSI-high: High levels of microsatellite instability
MSI-low: Low levels of MSI
MSS: Microsatellite-stable
NGS: Next-generation sequencing
SSA/P: Sessile serrated adenoma/polyp
